# Supplementary material for: A Candidate RNAi Screen Reveals Diverse RNA-Binding Protein Phenotypes in Drosophila Flight Muscle
Source: Cells. 2021 Sep 22;10(10):2505. doi: 10.3390/cells10102505 (PMC8534295; doi:10.3390/cells10102505)
Supplement: Supplementary file 1 [file cells-10-02505-s001.zip › cells-1348505-supplementary.pdf]

Supplemental Figure Legends

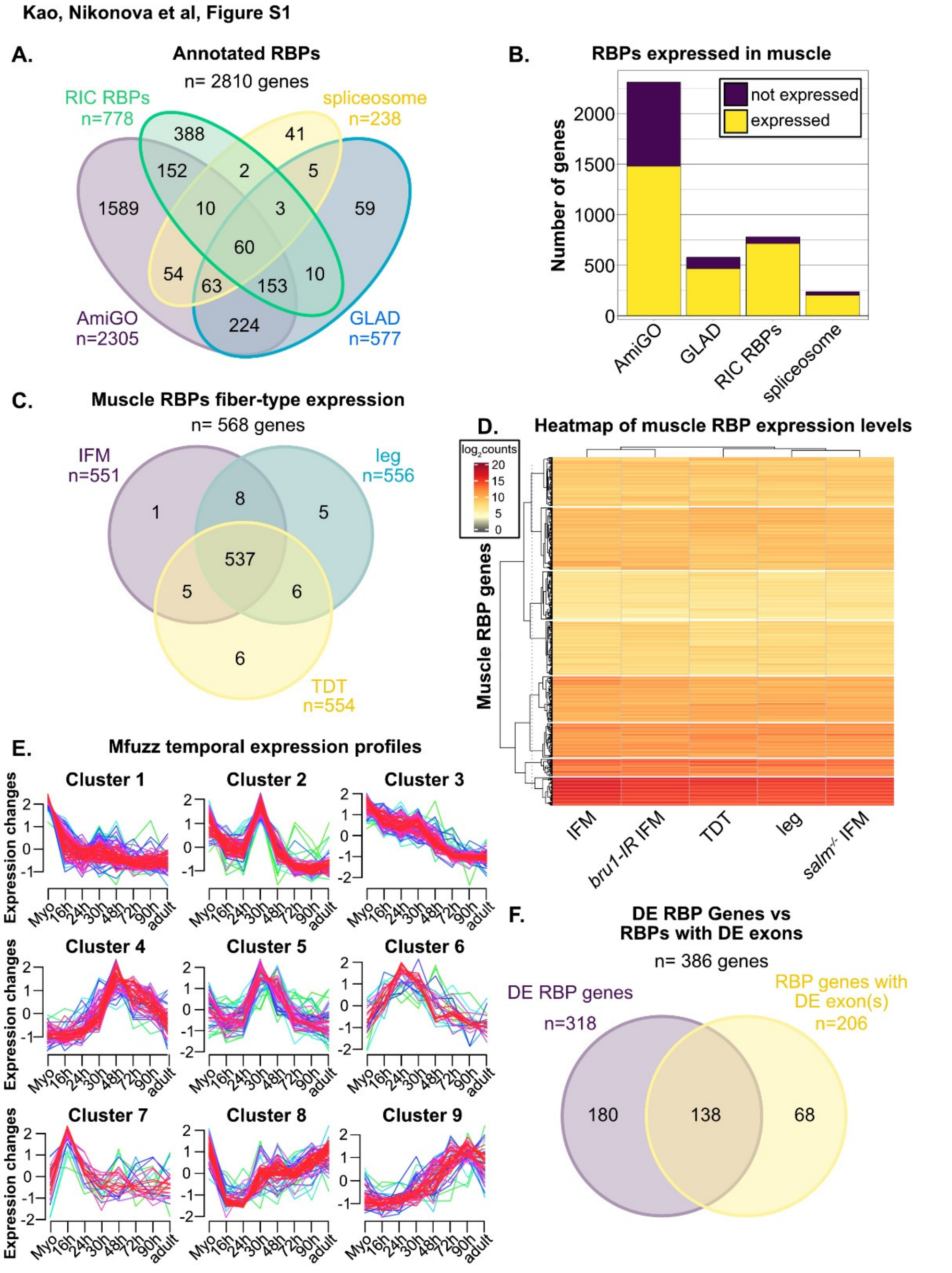

Figure S1. Dynamics of RBP expression in muscle. **(A)** Venn diagram illustrating the overlap between 4 different annotations of RBPs or spliceosome components, including "RNA binding" and "mRNA binding" terms from AmiGO (purple), RBPs in the GLAD database (cyan), RBPs identified by RNA interactome capture (RIC, green), and spliceosome components in Spliceosome Database (yellow). **(B)** Bar plot of the number of RBP genes in each annotation that are expressed (yellow, DESeq2 normalized counts > 100) in mRNA-Seq data from *Drosophila* muscle. **(C)** Venn diagram comparing expression of 568 muscle RBPs (expressed in muscle and identified in at least two annotations) between 1 d adult muscle fiber types (fibrillar IFM, purple; tubular TDT or jump muscle, yellow; tubular leg, cyan). Most RBPs are expressed in all muscle types. **(D)** Hierarchical clustering and heatmap of RBP expression (DESeq2 normalized counts) in IFM, leg and TDT as well as *bru1-IR* IFM and tubular-converted *salm*$^{-/-}$ IFM. Note that RBP expression levels vary widely and can be

higher or lower in different fiber types. **(E)** Mfuzz clustering of RBP temporal expression profiles in IFM. DESeq2 normalized count data from mRNA-Seq at 8 timepoints in IFM development were standard normalized and clustered into 9 distinct clusters. Traces in red represent genes with high cluster membership values (alpha > 0.9), while those in other colors reflect lower cluster membership values (alpha 0.5 – 0.9). RBPs display dynamic expression patterns during IFM development. **(F)** Venn diagram comparison of the number of differentially expressed (DE) RBP genes (purple) to the number of RBP genes with differential exon use (yellow) between fibrillar IFM and either tubular leg or TDT.

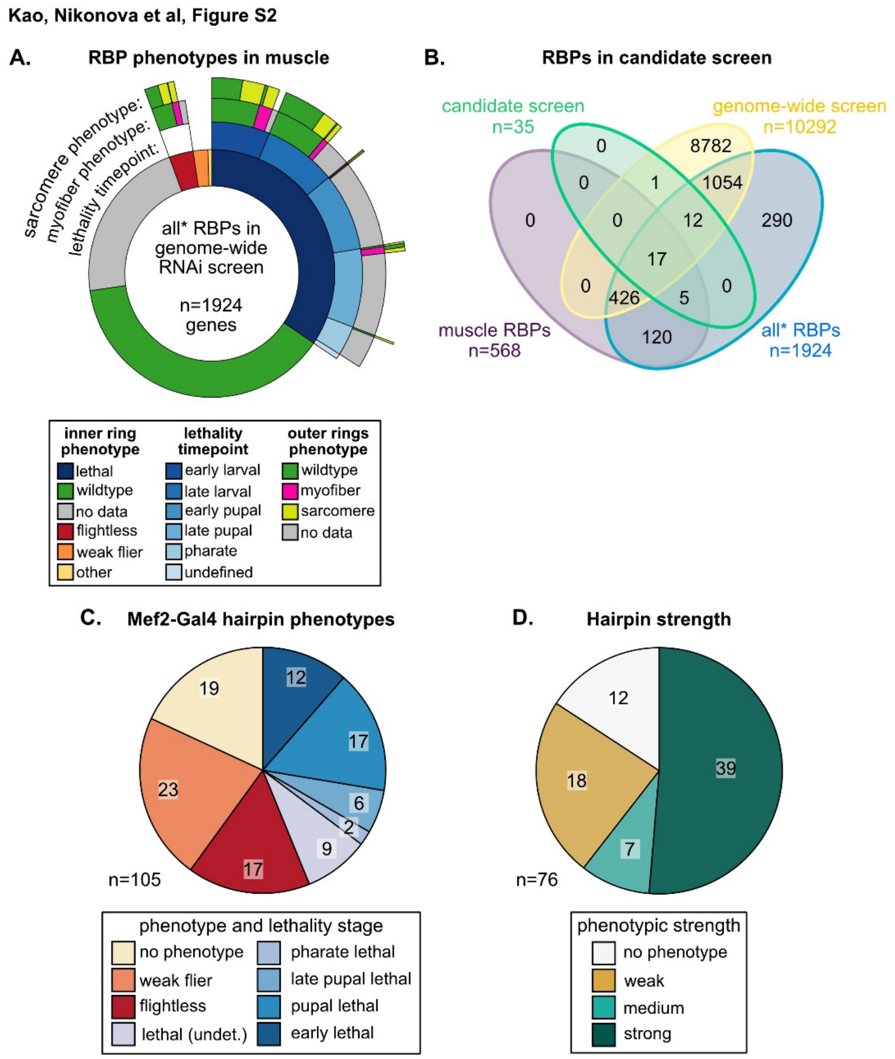

Figure S2. RBP phenotypes with Mef2-Gal4 mediated knockdown in *Drosophila* muscle. **(A)** Sunburst plot of all* RBP phenotypes in muscle from a published genome-wide RNAi screen (Schnorrer et al., 2010). * = "All" RBPs in this plot is defined as the 1924 RBP genes expressed in mRNA-Seq data from *Drosophila* muscle and annotated in any of the four annotations (AmiGO, GLAD, RIC or Spliceosome Database), and thus reflects a less conservative estimate than the 568 muscle RBPs defined in Figure 1 A. The inner ring summarizes lethality and flight phenotypes. The second ring

depicts the stage of lethality. The third and fourth rings summarize myofiber (magenta) and sarcomere (yellow) phenotypes, respectively. **(B)** Venn diagram of the 35 RBPs included in the candidate screen (green) and their overlap with muscle RBPs as defined in Figure 1 A (purple) versus all* RBPs from Figure S2 A (cyan). 5 of the RBPs we tested were not included in the original genome-wide screen (yellow). **(C)** Pie chart summary of RBP phenotypes for individual hairpins from our candidate screen when crossed to Mef2-Gal4. **(D)** Pie chart summary of the phenotypic strength of RNAi hairpins tested in this screen. Only 76 hairpins where more than one hairpin targeted the same gene were included. "Strength" was defined as "strong" if a hairpin line resulted in complete loss of flight or lethality, "medium" if > 50% of flies were flightless, "weak" if 20-50% of the flies were flightless and "no phenotype" if the line had wildtype flight ability.

Kao, Nikonova et al, Figure S3

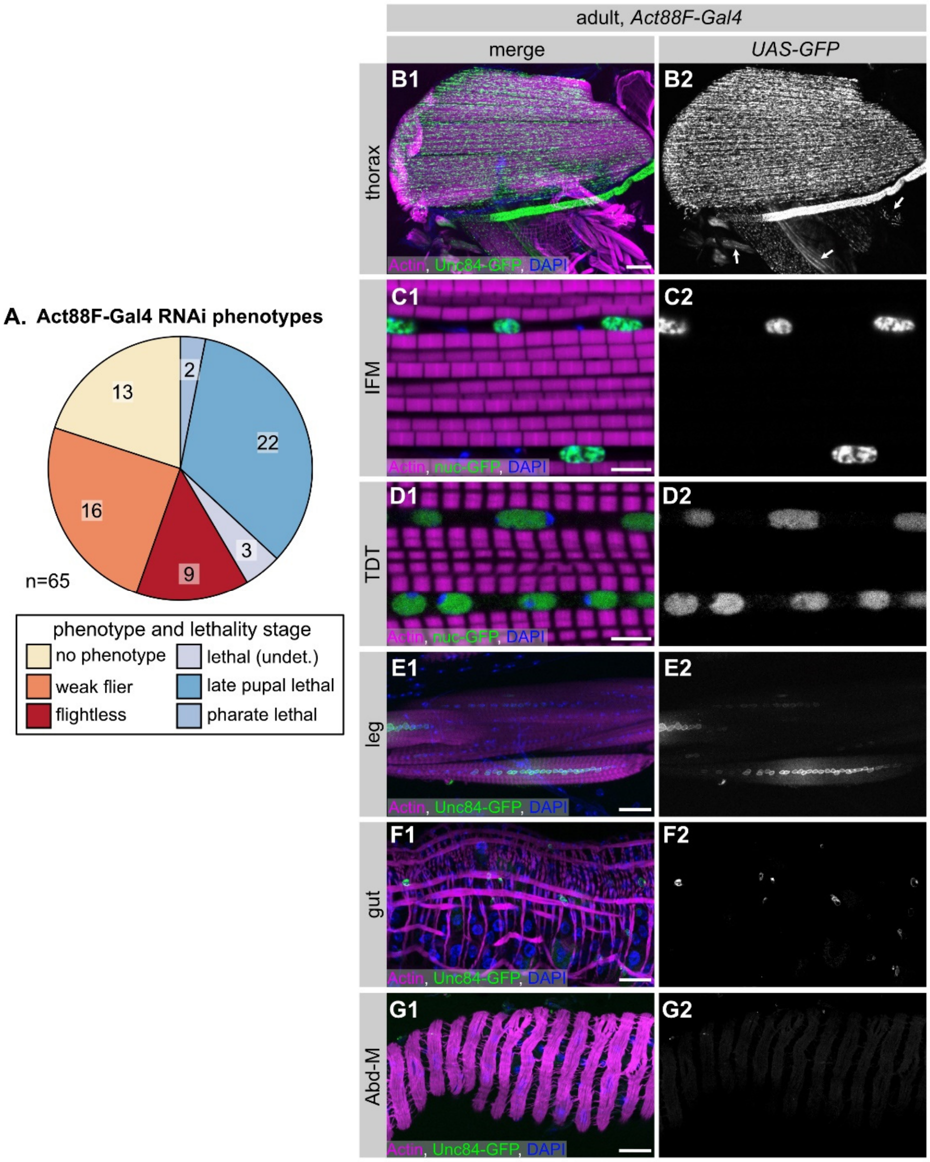

Figure S3. Act88F-Gal4 is expressed in a subset of tubular muscles. **(A)** Pie chart summary of RBP phenotypes for individual hairpins when crossed to Act88F-Gal4. **(B-G)** Confocal images from young adult flies of Act88F-Gal4 expression in different muscle types as detected by a nuclear localized UAS-Unc84-GFP or UAS-nuc-GFP reporter. High levels of GFP expression are restricted to IFM (**B1-C2**), but weaker levels of GFP expression can be observed in nuclei from jump muscle (TDT, **D1-2**), in specific legs muscles, typically in the upper leg segment (**E1-2**) and sporadically in gut muscle (**F1-2**). GFP was never observed in abdominal muscle (Abd-M, **G1-2**). As nuclear-localized GFP is very stable, we cannot distinguish if this is transient, developmental expression or continuous expression of the driver line.

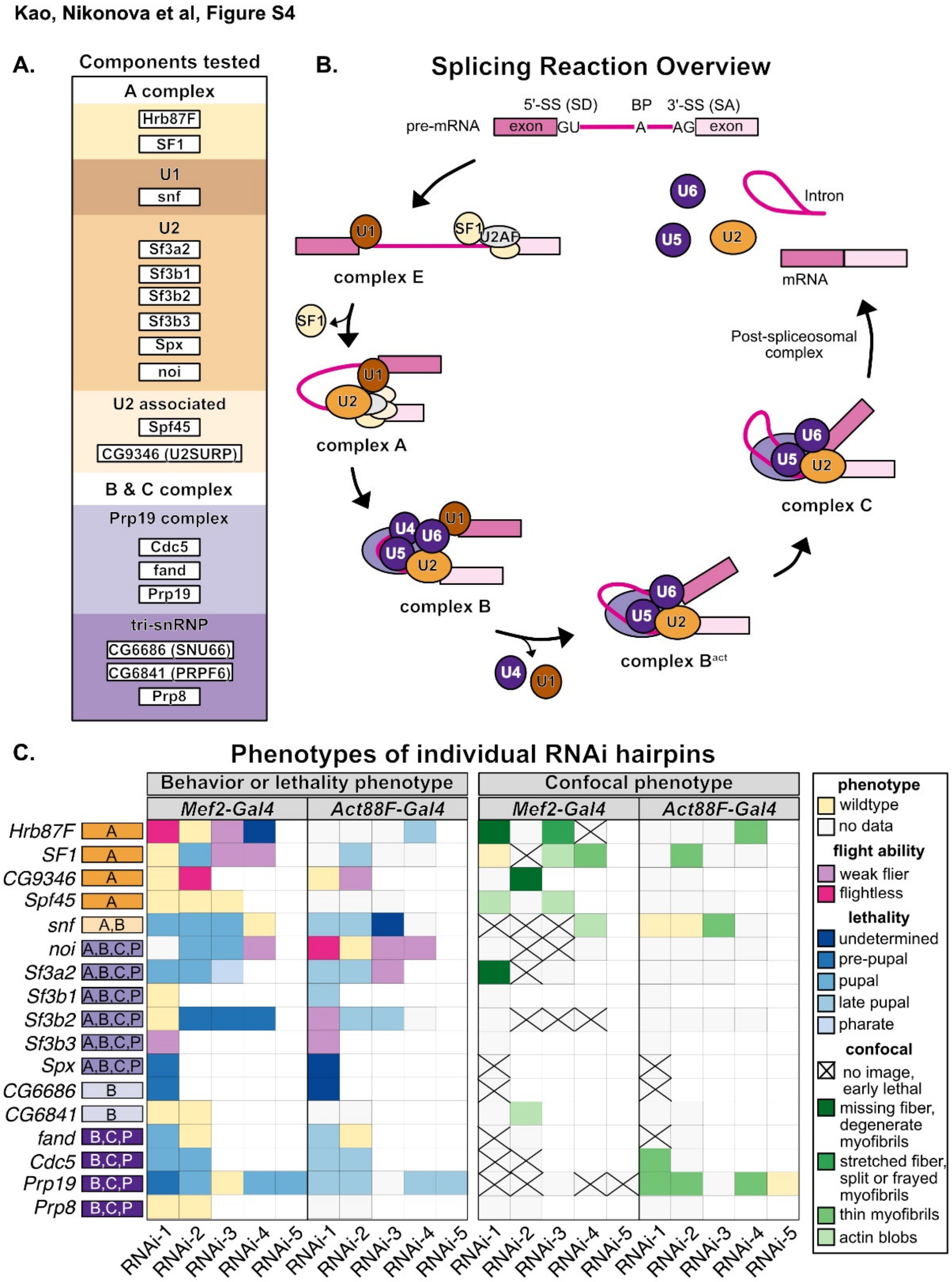

Figure S4. Knockdown of spliceosome components produces muscle-specific phenotypes. **(A)** Schematic of spliceosome components included in the screen and their association to described spliceosome complexes including the A complex (yellow), U1 complex (brown), U2 complex (orange), U2 associated factors (light orange), Prp19 complex (light purple) and tri-snRNP complex (purple) as annotated in the Spliceosome Database (Cvitkovic and Jurica, 2013). **(B)** Overview schematic of the splicing reaction. The upstream exon (pink) ends with the 5′-SS (splice site), also referred to as the splice donor (SD), while the downstream exon (light pink) begins with the 3′-SS, also referred to as the splice acceptor (SA). U1 associates at the 5′-SS while A-complex component SF1 binds the branch point (BP) sequence and U2AF together with other factors, including the hnRNP-A family RBP Hrb87F, bind at the 3′-SS to form the E complex. U2 assembles on the 3′-SS and U1 and U2 associate forming the A-complex. The Prp19 and tri-snRNP complexes assemble with U1 and U2 forming the B complex, which is activated as U1 and U4 are released. The two-step transesterification reaction to remove the intron and join the exons is accomplished as the catalytic core transitions through the C complex and ultimately dissociates. **(C)** Heatmap-style plot of lethality, flight ability and confocal phenotype for all spliceosome genes and hairpins tested in the screen. Spliceosome association of each gene is listed and colored as in Figure 2 A. For all surviving adults, RNAi lines were scored as wildtype (WT, light yellow), weak flier (purple) or flightless (magenta). For lines with no surviving adults, lethality stage was scored as pre-pupal, pupal, late pupal, pharate or undetermined (shades of blue). Muscle phenotypes were evaluated by confocal microscopy in young adults or 90 h APF pupae. Lethal lines where pupae died before 90 h APF and could not be imaged are marked with an "X". Lines where data is unavailable are colored light gray. Confocal phenotypes are summarized from weakest to strongest as Zebra bodies or actin blobs (light green), thin myofibrils (green), stretched myofibers or split or frayed myofibrils (medium green) and missing fibers or degenerate myofibrils and sarcomeres (dark green). Note that phenotypes for B and C complex components tend to be stronger and that phenotypic severity for different hairpins targeting the same gene can vary, likely reflecting differences in knockdown efficiency.

Kao, Nikonova et al, Figure S5

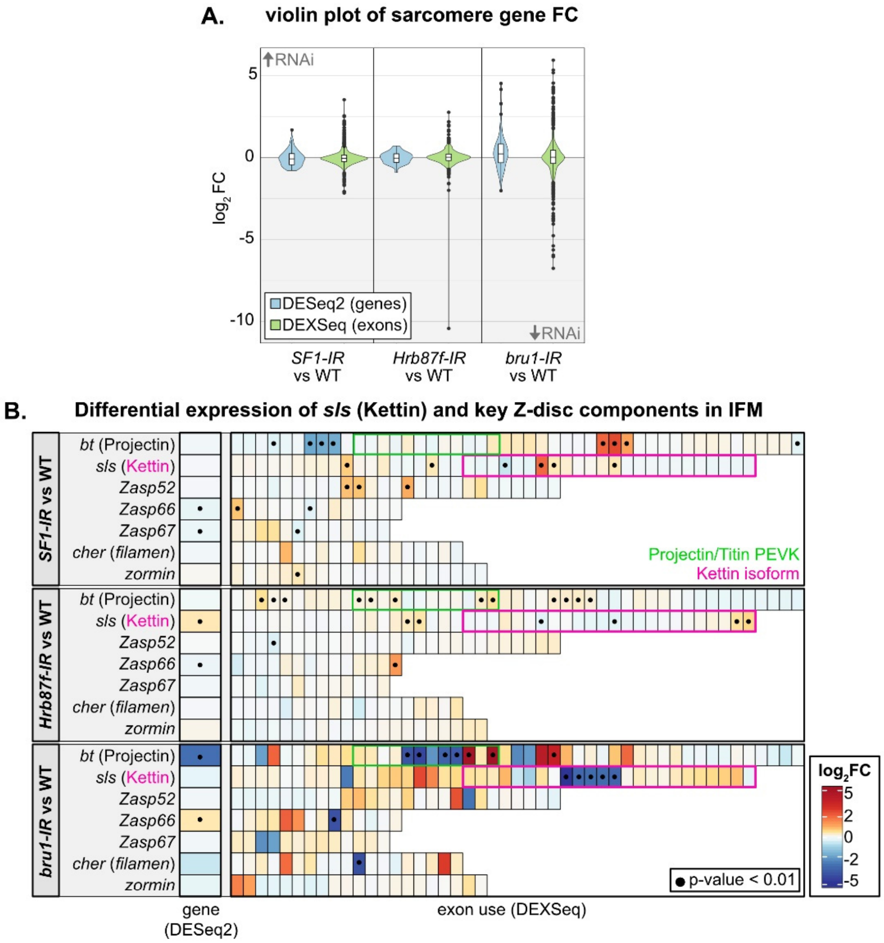

Figure S5. *SF1-IR* and *Hrb87F-IR* cause changes in gene expression and exon use in z-disc components. **(A)** Violin plot of log2FC values for all sarcomere genes (blue) and sarcomere gene exons (green) in *SF1-IR*, *Hrb87F-IR*, and *bru1-IR*. Gray shaded region denotes decreased expression in the knockdown condition. Fold change values have a broader distribution at the level of exon use, reflecting the function of SF1, Hrb87F and Bru1 in splicing. **(B)** Heatmap of gene and exon log2FC values in *SF1-IR*, *Hrb87F-IR*, and *bru1-IR* knockdown IFM at 72 h APF for z-disc components that directly bind or influence the localization of Kettin, including *bt* (which encodes the Titin-like protein Projectin), *sls* (which encodes the Titin-related Sallimus and Kettin protein isoforms), *Zasp52*, *Zasp66*, *Zasp67* (Zasp proteins contribute to z-disc integrity and regulate myofibril width), *cher* (an F-actin binding filamin family protein) and *zormin*. The PEVK region of *bt* that is differentially spliced between fiber-types and implicated in passive stiffness is outlined in green, and Kettin encoding exons in *sls* are outlined in magenta. A dot denotes genes with a DESeq2 adjusted p-value < 0.01 and exons with a DEXSeq p-value < 0.01.

Kao, Nikonova et al, Figure S6

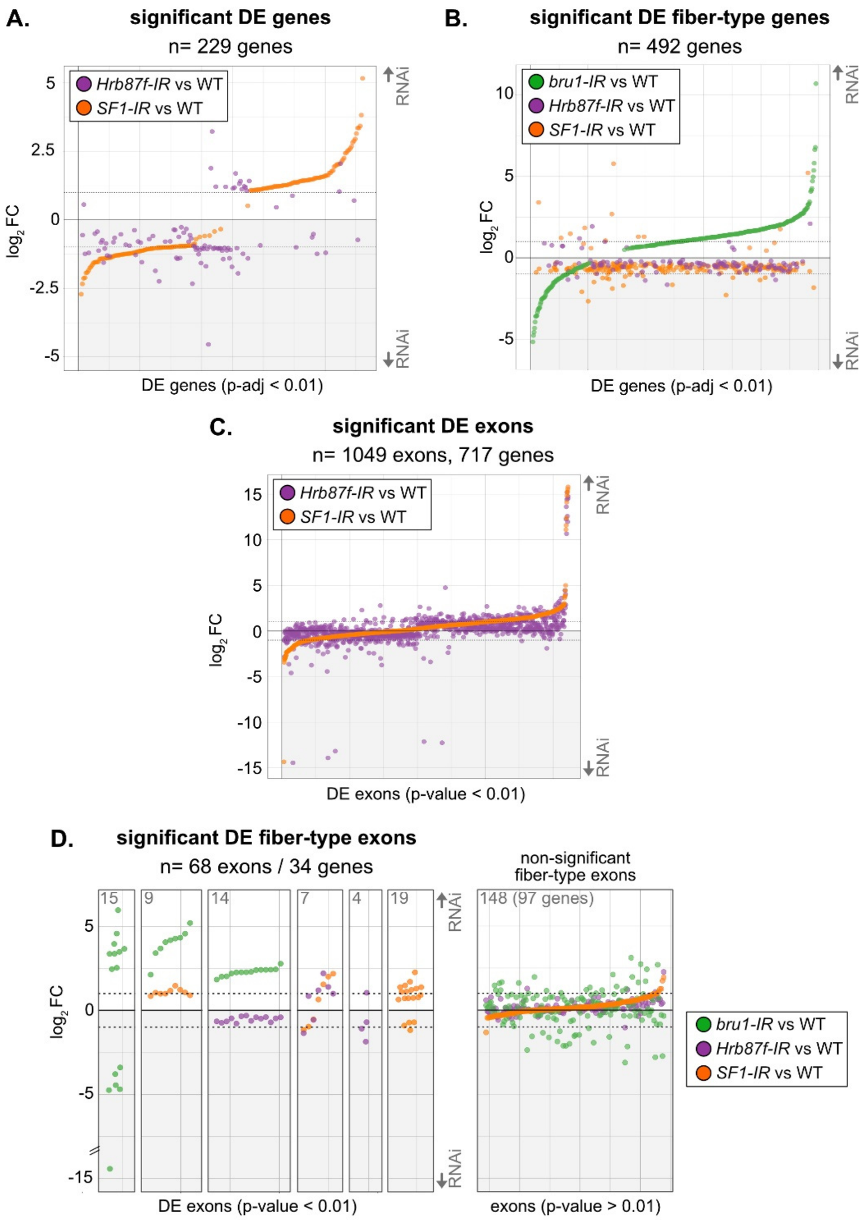

Figure S6. SF1 and Hrb87F do not regulate fiber-type specific gene expression or exon use. **(A)** Plot of log_2FC values for all 229 significantly differentially expressed (DE) genes (DESeq2 adjusted *p*-value < 0.01) in *SF1-IR* (orange) or *Hrb87F-IR* (purple). **(B)** Plot of log_2FC values for 492 fiber-type specific genes significantly differentially expressed (DESeq2 adjusted *p*-value < 0.01) in either *bru1-IR* (green), *SF1-IR* (orange) or *Hrb87F-IR* (purple). Fiber-type specific genes are significantly regulated (DESeq2 adjusted p-value < .01, abs(log_2FC) > 1.5) in the same direction in 1 d adult *salm*$^{-/-}$ IFM, leg and jump muscle (TDT) samples as compared to WT IFM (Spletter et al., 2015). As compared to Bru1, SF1 and Hrb87F do not impact fiber-type specific gene expression. **(C)** Plot of log_2FC values for all 1049 significantly differentially expressed (DE) exons (DEXSeq p-value < 0.01) in *SF1-IR* (orange) or *Hrb87F-IR* (purple). **(D)** Plot of log_2FC values for 68 significantly DE fiber-type specific exons (DEXSeq p-value < 0.01) (*bru-IR* exons, green; *Hrb87F-IR* exons, purple; *SF1-IR* exons, orange). The number of exons is noted in the top left of each plot region. Fiber-type specific exons are

significantly regulated (DEXSeq p-value < 0.01) in the same direction in 1 d adult *salm*$^{-/-}$ IFM, leg and jump muscle (TDT) samples as compared to WT IFM (Spletter et al., 2015). Note the distinct regulatory dynamics for individual exons between *bru1-IR* and *SF1-IR* or *Hrb87F-IR*, as well as the 148 exons that are not significantly regulated by Bru1, SF1 or Hrb87F. In all plots, dotted lines mark a log_2FC value of 1 and -1. Gray shaded region denotes genes downregulated in the knockdown condition.

Supplemental Table Legends

Table S1. Key Resources Table. This table includes a list of the sources and identifiers for genetic and chemical reagents used in this manuscript, as well as the references and sources for bioinformatic packages used in mRNA-Seq data analysis.

Gene	Gene_tested	Hairpin_ID	Stock Center	Stock_Number	SYMBOL	ANNOTATION_SYMBOL
orb	orb	TRiP.JF01864	Bloomington	25843	orb	CG10868
Rbfox1,_A2BP1	Rbfox1	TRiP.JF02600	Bloomington	27286	Rbfox1	CG32062
SF1	SF1	TRiP.JF02871	Bloomington	28036	SF1	CG5836
me31B	me31B	TRiP.HM05052	Bloomington	28566	me31B	CG4916
Rbp9	Rbp9	TRiP.JF03084	Bloomington	28669	Rbp9	CG3151
mbl	mbl	TRiP.JF03264	Bloomington	29585	mbl	CG33197
mbl	mbl	TRiP.JF03264	Bloomington	29585	mbl	CG33197
Hrb87F,_hnRNP_A	Hrb87F	TRiP.JF01757	Bloomington	31244	Hrb87F	CG12749
Rm62	Rm62	TRiP.JF01385	Bloomington	31395	Rm62	CG10279
Hrb87F,_hnRNP_A	Hrb87F	TRiP.JF01250	Bloomington	31472	Hrb87F	CG12749
Rbfox,_A2BP1	Rbfox1	TRiP.HMS00478	Bloomington	32476	Rbfox1	CG32062
Prp19	Prp19	TRiP.HMS00652	Bloomington	32865	Prp19	CG5519
Sf3b1	Sf3b1	TRiP.HMS00055	Bloomington	33650	Sf3b1	CG2807
Sf3b2	Sf3b2	TRiP.HMS00056	Bloomington	33651	Sf3b2	CG3605
me31B	me31B	TRiP.HMS00539	Bloomington	33675	me31B	CG4916
Sf3b3	Sf3b3	TRiP.HMS00614	Bloomington	33731	Sf3b3	CG13900
Prp8	Prp8	TRiP.HMS01297	Bloomington	34622	Prp8	CG8877
YT521-B	YT521-B	TRiP.HMS01302	Bloomington	34627	Ythdc1	CG12076
Rm62	Rm62	TRiP.HMS00144	Bloomington	34829	Rm62	CG10279
noi,_Sf3a3	noi	TRiP.HMS00163	Bloomington	34845	noi	CG2925
mub	mub	TRiP.HMS00189	Bloomington	34872	mub	CG7437
mub	mub	TRiP.HMS00189	Bloomington	34873	mub	CG7437
mub	mub	TRiP.HMS00189	Bloomington	34875	mub	CG7437
Atx2	Atx2	TRiP.HMS01392	Bloomington	36114	Atx2	CG5166
me31B	me31B	TRiP.GL00695	Bloomington	38923	me31B	CG4916

Ime4/Mettl 3	Mettl3	TRiP.GL01126	Bloomington	41590	Mettl3	CG5933
Rbm17,_Spf 45	Rbm17	TRiP.HMS0235 1	Bloomington	41954	Spf45	CG17540
Rbp9	Rbp9	TRiP.GL01167	Bloomington	42796	Rbp9	CG3151
orb	orb	TRiP.GL01484	Bloomington	43143	orb	CG10868
Atx2	Atx2	TRiP.HMS0272 6	Bloomington	44012	Atx2	CG5166
Bru1,_Aret	Bru1	TRiP.HMC023 74	Bloomington	44483	bru1	CG31762
Cdc5	Cdc5	TRiP.HMC029 18	Bloomington	44524	Cdc5	CG6905
doa	doa	TRiP.HMJ0312 1	Bloomington	50903	Doa	CG42320
snf,_SNRP A	snf	TRiP.HMC031 97	Bloomington	51459	snf	CG4528
Prp6_(CG6 841)	Prp6	TRiP.HMC034 84	Bloomington	51909	Prp6	CG6841
Hrb87F,_hn RNP_A	Hrb87F	TRiP.HMC036 79	Bloomington	52937	Hrb87F	CG12749
SF1	SF1	TRiP.HMC036 80	Bloomington	52938	SF1	CG5836
Smn	Smn	TRiP.HMC038 32	Bloomington	55158	Smn	CG16725
Sart1_(CG6 686)	Sart1	TRiP.HMC039 13	Bloomington	55202	Sart1	CG6686
Cdc5	Cdc5	TRiP.HMC039 48	Bloomington	55261	Cdc5	CG6905
Sf3a2	Sf3a2	TRiP.HMC037 99	Bloomington	55650	Sf3a2	CG10754
How	How	TRiPHMC0382 0	Bloomington	55665	how	CG10293
snf,_SNRP A	snf	TRiP.HMC042 00	Bloomington	55914	snf	CG4528
U2SURP_(CG9346)	U2SURP	TRiP.HMC041 19	Bloomington	56898	U2SURP	CG9346
Spx,_Sf3b4	Spx	TRiP.HMJ2387 5	Bloomington	62421	Spx	CG3780
Mettl14	Mettl14	TRiP.HMC055 66	Bloomington	64547	Mettl14	CG7818
Ime4/Mettl 3	Mettl3	TRiP.HMS0601 1	Bloomington	80431	Mettl3	CG5933
Ime4/Mettl 3	Mettl3	TRiP.HMS0602 8	Bloomington	80448	Mettl3	CG5933
Ime4/Mettl 3	Mettl3	TRiP.HMS0603 0	Bloomington	80450	Mettl3	CG5933
Ime4/Mettl 3	Mettl3	5933R	NIG	NIG5933R -4	Mettl3	CG5933
Hrb87F,_hn RNP_A	Hrb87F	KK108162	Vienna Drosophila Re-source Center (VDRC)	100732	Hrb87F	CG12749
Rbp9	Rbp9	KK109093	Vienna Drosophila Re-source Center (VDRC)	101412	Rbp9	CG3151
doa	doa	KK111879	Vienna Drosophila Re-source Center (VDRC)	102520	Doa	CG42320
sbr	sbr	KK100882	Vienna Drosophila Re-source Center (VDRC)	103715	sbr	CG1664
fand	fand	KK101751	Vienna Drosophila Re-source Center (VDRC)	104186	fand	CG6197

snf,_SNRP A	snf	KK107649	Vienna Drosophila Re-source Center (VDRC)	104334	snf	CG4528
noi,_Sf3a3	noi	KK101842	Vienna Drosophila Re-source Center (VDRC)	105354	noi	CG2925
Mettl14	Mettl14	KK102058	Vienna Drosophila Re-source Center (VDRC)	105434	Mettl14	CG7818
mbl	mbl	KK107778	Vienna Drosophila Re-source Center (VDRC)	105486	mbl	CG33197
mbl	mbl	KK107778	Vienna Drosophila Re-source Center (VDRC)	105486	mbl	CG33197
Sf3b2	Sf3b2	KK101239	Vienna Drosophila Re-source Center (VDRC)	105639	Sf3b2	CG3605
Bru1,_Aret	Bru1	KK110026	Vienna Drosophila Re-source Center (VDRC)	107459	bru1	CG31762
Prp19	Prp19	KK101335	Vienna Drosophila Re-source Center (VDRC)	108575	Prp19	CG5519
Atx2	Atx2	KK100423	Vienna Drosophila Re-source Center (VDRC)	108843	Atx2	CG5166
Rm62	Rm62	KK112269	Vienna Drosophila Re-source Center (VDRC)	110102	Rm62	CG10279
Rbfox1,_A2 BP1	Rbfox1	KK109100	Vienna Drosophila Re-source Center (VDRC)	110518	Rbfox1	CG32062
SF1	SF1	GD5176	Vienna Drosophila Re-source Center (VDRC)	13425	SF1	CG5836
SF1	SF1	GD5176	Vienna Drosophila Re-source Center (VDRC)	13426	SF1	CG5836
How	How	GD13756	Vienna Drosophila Re-source Center (VDRC)	1462	how	CG10293
Prp8	Prp8	GD6578	Vienna Drosophila Re-source Center (VDRC)	18565	Prp8	CG8877
doa	doa	GD8588	Vienna Drosophila Re-source Center (VDRC)	19066	Doa	CG42320
doa	doa	GD8588	Vienna Drosophila Re-source Center (VDRC)	20120	Doa	CG42320
noi,_Sf3a3	noi	GD9855	Vienna Drosophila Re-source Center (VDRC)	20943	noi	CG2925
noi,_Sf3a3	noi	GD9855	Vienna Drosophila Re-source Center (VDRC)	20945	noi	CG2925
Ime4/Mettl 3	Mettl3	GD9882	Vienna Drosophila Re-source Center (VDRC)	20968	Mettl3	CG5933
doa	doa	GD10146	Vienna Drosophila Re-source Center (VDRC)	21294	Doa	CG42320
Prp19	Prp19	GD11681	Vienna Drosophila Re-source Center (VDRC)	22146	Prp19	CG5519
Prp19	Prp19	GD11681	Vienna Drosophila Re-source Center (VDRC)	22147	Prp19	CG5519
Sf3b2	Sf3b2	GD11027	Vienna Drosophila Re-source Center (VDRC)	26250	Sf3b2	CG3605
Sf3b2	Sf3b2	GD11027	Vienna Drosophila Re-source Center (VDRC)	26252	Sf3b2	CG3605
U2SURP_(CG9346)	U2SURP	GD14191	Vienna Drosophila Re-source Center (VDRC)	27013	U2SURP	CG9346
mbl	mbl	GD13374	Vienna Drosophila Re-source Center (VDRC)	28732	mbl	CG33197
mbl	mbl	GD13374	Vienna Drosophila Re-source Center (VDRC)	28732	mbl	CG33197
salm	salm	GD1525	Vienna Drosophila Re-source Center (VDRC)	3029	salm	CG6464

Sf3a2	Sf3a2	GD7087	Vienna Drosophila Resource Center (VDRC)	31346	Sf3a2	CG10754
Sf3a2	Sf3a2	GD7087	Vienna Drosophila Resource Center (VDRC)	31347	Sf3a2	CG10754
sbr	sbr	GD9182	Vienna Drosophila Resource Center (VDRC)	32690	sbr	CG1664
sbr	sbr	GD9182	Vienna Drosophila Resource Center (VDRC)	32691	sbr	CG1664
Rbm17,_Spf45	Rbm17	GD9411	Vienna Drosophila Resource Center (VDRC)	32948	Spf45	CG17540
Rbm17,_Spf45	Rbm17	GD9411	Vienna Drosophila Resource Center (VDRC)	32949	Spf45	CG17540
snf,_SNRPA	snf	VSH330329	Vienna Drosophila Resource Center (VDRC)	330329	snf	CG4528
YT521-B	YT521-B	VSH330558	Vienna Drosophila Resource Center (VDRC)	330558	Ythdc1	CG12076
Prp6_(CG6841)	Prp6	GD10654	Vienna Drosophila Resource Center (VDRC)	34254	Prp6	CG6841
How	How	KK100775	Vienna Drosophila Resource Center (VDRC)	35810	how	CG10293
How	How	KK100775	Vienna Drosophila Resource Center (VDRC)	37342	how	CG10293
Prp19	Prp19	GD11681	Vienna Drosophila Resource Center (VDRC)	41438	Prp19	CG5519
Bru1,_Aret	Bru1	GD8699	Vienna Drosophila Resource Center (VDRC)	41568	bru1	CG31762
clu	clu	CG13926	Vienna Drosophila Resource Center (VDRC)	42136	clu	CG8443
clu	clu	GD13926	Vienna Drosophila Resource Center (VDRC)	42138	clu	CG8443
Hrb59	rump	GD14202	Vienna Drosophila Resource Center (VDRC)	44659	rump	CG9373
sbr	sbr	GD14541	Vienna Drosophila Resource Center (VDRC)	46117	sbr	CG1664
fand	fand	GD11858	Vienna Drosophila Resource Center (VDRC)	46312	fand	CG6197
doa	doa	GD16021	Vienna Drosophila Resource Center (VDRC)	46449	Doa	CG42320
Rm62	Rm62	GD17219	Vienna Drosophila Resource Center (VDRC)	46908	Rm62	CG10279
Bru1,_Aret	Bru1	GD17093	Vienna Drosophila Resource Center (VDRC)	48237	bru1	CG31762
Mettl14	Mettl14	GD16300	Vienna Drosophila Resource Center (VDRC)	48560	Mettl14	CG7818
me31B	me31B	GD11470	Vienna Drosophila Resource Center (VDRC)	49378	me31B	CG4916

Table S2. Expression data of muscle RBPs. This spreadsheet contains the data used to generate plots in Figures 1, S1, 2 and S2. It includes a list of all RBPs contained in different annotations, those RBPs we categorize as "muscle RBPs" and a list of expression values for those genes in muscle. Standard normalized expression values used for Mfuzz clustering as well as cluster membership and core cluster expression profiles are provided. A list of both differentially expression RBPs and RBP exons used for hierarchical clustering and heatmaps is included.

Tab Name	Figure	Description
Sunburst	Figure 1F, S2A	table includes counts data plotting in the sunburst diagrams of the number of RBP genes in each phenotypic cateogry
anno-tated_RBPs	Figure1, S1	list of all RBPs included in the 4 annotations: AmiGO, RIC, GLAD and SpliceosomeDatabase
MuscleEx-pressedRBPs	Figure1, S1, S2	table of gene identifiers for all 568 genes identified by at least 2 RBP annotations and expressed in muscle; includes DESeq2 nor-malized counts values used to generate heatmaps
Mfuzz_input	Figure 1B, S1E	table of normalized expression counts and standard normalized values used as input for 3 rounds of Mfuzz clustering
Mfuzz_clus-ters	Figure S1E	table of genes, cluster association and cluster membership values (alpha) for Mfuzz temporal expression profile clustering
Mfuzz_corep rofiles	Figure 1B	average expression profiles and number of genes in each Mfuzz cluster, used for plotting temporal expression heatmap
Venn_DER-BPs	Figure S1F	genes in VennDiagram regions for differentially expressed RBPs between IFM and tubular leg, TDT and salm-/- IFM samples com-pared to RBP genes with DE exons between those samples
heatmap_log 2FC_exons	Figure 1C	table of exon log2FC values plotted in the heatmap in Figure 1D and corresponding gene identifiers
heatmap_log 2FC_genes	Figure 1D	table of log2FC values plotted in the heatmap in Figure 1C and corresponding gene identifiers

Table S3. Data from candidate RBP screen. This table includes data used to generate the plots in Figures 2-5 and summarizing results from the various assays included in our RBP screen. It includes a list of all 35 genes tested, summarized data from lethality, flight and confocal assays and raw flight test data including number of flies tested.

Tab Name	Figure	Description
screenRBPs_ hairpins	Figure2-5	identifier information for all RNAi hairpins targeting RBPs used in this manuscript
geneinfo	Figure2-5	list of 35 genes targeted by hairpins in the RBP screen, includes identifiers and summary of gene data
screen_data	Figure2-5	summarized data of screen data for all hairpins including flight index and confocal phenotypes
FigureS5	Fig-ureS5C	table of data represented in the heatmap of phenotypes for spliceosome components
rawdata	Figure2-5	raw data of flight test values for each hairpin, including N flies tested for each line

Tab Name	Figure	Description
DESeq2_alldata	Figures 7&8, S5, S6	full data table from DESeq2 analysis of differential gene expression in SF1-IR, Hrb87F-IR and bru1-IR
DEXSeq_alldata	Figures 7&8, S5, S6	full data table from DEXSeq analysis of differential exon use in SF1-IR, Hrb87F-IR and bru1-IR
z-disc components	Figure S5	data table of expression values used in the heatmap to compare expression of z-disc components in SF1-IR, Hrb87F-IR and bru1-IR
Degenes	Figures 7&8, S5, S6	List of gene symbols for significant genes at different threshold levels used for GO enrichment analysis
Deexons	Figures 7&8, S5, S6	List of gene symbols for genes with significant exon use changes at different threshold levels used for GO enrichment analysis
DESeq2_GOBP	Figure 7	List of GO biological process terms for DE genes after rrvgo reduction; original GO analysis performed in GOrilla
DEXSeq_GOBP	Figure 7	List of GO biological process terms for genes with DE exons after rrvgo reduction; original GO analysis performed in GOrilla
DESeq2_fibertypegenes	Figure S6	Genes with differential expression (DESeq2) between fiber-types in Drosophila (IFM vs salm-/- IFM AND (IFM vs leg OR IFM vs TDT)), table lists DE gene expression log2FC values in in SF1-IR, Hrb87F-IR and bru1-IR
DEXSeq_fibertypeexons	Figure S6	Exons with differential use (DEXSeq) between fiber-types in Drosophila (IFM vs salm-/- IFM AND (IFM vs leg OR IFM vs TDT)), table lists DE exon use log2FC values in in SF1-IR, Hrb87F-IR and bru1-IR

Table S4. mRNA-Seq data from *SF1-IR* and *Hrb87F-IR*. This table lists data related to the bioinformatic analysis of mRNA-Seq data from *SF1-IR* and *Hrb87F-IR* and *bru1-IR* in IFM dissected from 72 h APF pupae. Full data tables of DESeq2 and DEXSeq analysis are provided, as well as expression data for select z-disc proteins. Lists of gene symbols used to perform GO analysis of biological process terms as well as the resulting lists of terms are included. Lists of genes and exons differentially regulated between tubular and fibrillar muscle types as well as their log2FC values in *SF1-IR* and *Hrb87F-IR* and *bru1-IR* are also provided.
